# A Gadolinium(III) Complex Based on the Thymine Nucleobase with Properties Suitable for Magnetic Resonance Imaging

**DOI:** 10.3390/ijms22094586

**Published:** 2021-04-27

**Authors:** Marta Orts-Arroyo, Amadeo Ten-Esteve, Sonia Ginés-Cárdenas, Isabel Castro, Luis Martí-Bonmatí, José Martínez-Lillo

**Affiliations:** 1 Instituto de Ciencia Molecular (ICMol), Universitat de València, c/Catedrático José Beltrán 2, Paterna, 46980 Valencia, Spain; marta.orts-arroyo@uv.es (M.O.-A.); isabel.castro@uv.es (I.C.); 2 Radiology Department and Biomedical Imaging Research Group (GIBI230), La Fe University and Polytechnic Hospital and La Fe Health Research Institute, 46026 Valencia, Spain; ten_ama@gva.es (A.T.-E.); soniagibi230@gmail.com (S.G.-C.)

**Keywords:** thymine, gadolinium, contrast agent, magnetic resonance, metal complexes, crystal structure, relaxivity

## Abstract

The paramagnetic gadolinium(III) ion is used as contrast agent in magnetic resonance (MR) imaging to improve the lesion detection and characterization. It generates a signal by changing the relaxivity of protons from associated water molecules and creates a clearer physical distinction between the molecule and the surrounding tissues. New gadolinium-based contrast agents displaying larger relaxivity values and specifically targeted might provide higher resolution and better functional images. We have synthesized the gadolinium(III) complex of formula [Gd(thy)_2_(H_2_O)_6_](ClO_4_)_3_·2H_2_O (**1**) [thy = 5-methyl-1H-pyrimidine-2,4-dione or thymine], which is the first reported compound based on gadolinium and thymine nucleobase. **1** has been characterized through UV-vis, IR, SEM-EDAX, and single-crystal X-ray diffraction techniques, and its magnetic and relaxometric properties have been investigated by means of SQUID magnetometer and MR imaging phantom studies, respectively. On the basis of its high relaxivity values, this gadolinium(III) complex can be considered a suitable candidate for contrast-enhanced magnetic resonance imaging.

## 1. Introduction

Thymine is one of the four natural nitrogen bases that are precursors and part of the structure of the deoxyribonucleic acid (DNA) macromolecule [1,2]. This pyrimidine base has been widely studied, in part because of the common mutations of DNA caused when adjacent thymines are irradiated by UV light and are dimerized, generating the well-known thymine dimers [2,3]. Furthermore, considerable effort has also been devoted to the rational design of drugs that might selectively inhibit thymine biosynthesis, thereby blocking DNA replication, especially in rapidly dividing malignant cells [2].

In comparison with other natural nucleobases, the coordination chemistry of thymine-based metal complexes has been much less investigated. Most of the thymine-containing complexes have been prepared with the nucleobase in the form of thyminate anion, that is, releasing one or two protons of its N-H groups, whereas the reported examples obtained with the thymine molecule acting through its carbonyl groups as a neutral ligand toward the metal are much scarcer [4,5,6,7]. In that respect, theoretical studies have been performed on metal clusters to investigate the preferential binding sites of the thymine molecule [8].

There exist some published thymine-based complexes that exhibit singular properties, as, for instance, the Ru^II^-thymine complex [Ru(PPh_3_)_2_(thy)(bpy)]PF_6_ [where PPh_3_ = triphenylphosphine and bpy = 2,2′-bipyridine], which is a potent cytotoxic agent with the ability to bind to DNA, inducing apoptotic cell death in human colon carcinoma [6,7]. Thymine has also shown to be a highly specific ligand toward Hg^II^ metal ion [9,10]. The discovery of the linear thymine-Hg^II^-thymine structure, which affords a high stabilization of thymine-thymine pairs in DNA, has led to the designing and development of recent thymine-based sensors [11,12,13].

Regarding lanthanide complexes, only two structures based on thyminate anion have been published so far, namely, the heteropolynuclear complexes of general formula [{Cp*Rh^III^(µ-thym)}_4_{Ln^III^(NO_3_)_2_}]^+^, where Ln = Dy^III^ and Er^III^, which form cationic metallacalix[4]arene-type systems [14]. Hence, no crystal structure of gadolinium complex based on thymine has been reported up to date.

**Figure 1:**
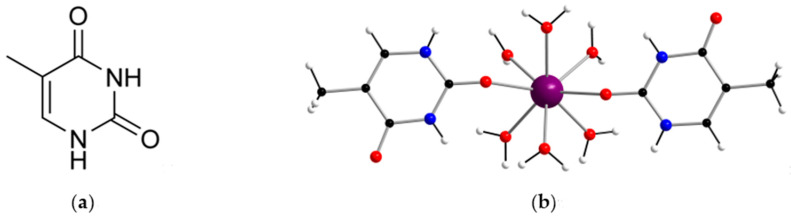
(**a**) Molecular structure of the thymine nucleobase (thy); (**b**) Mononuclear [Gd(thy)_2_(H_2_O)_6_]^3+^ complex in **1**. ClO_4_^−^ anions and non-coordinated water molecules have been omitted for clarity. Color code: purple, Gd; red, O; blue, N; black, C; white, H.

As a continuation of our interest in investigating biomolecule-based complexes [15,16,17,18,19,20], herein we report the synthesis and characterization of a new gadolinium(III) complex of formula [Gd(thy)_2_(H_2_O)_6_](ClO_4_)_3_·2H_2_O (**1**) [thy = thymine], which exhibits a linearly disposed thymine-Gd^III^-thymine structure (Figure 1 and Figure S1). **1** displays the first reported crystal structure based on gadolinium and thymine and is a suitable candidate for contrast-enhanced magnetic resonance (MR) imaging applications.

## 2. Results and Discussion

### 2.1. Synthetic Procedure

**Figure 2:**
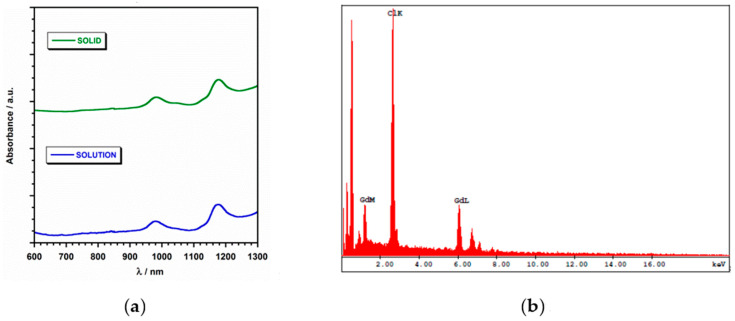
(**a**) Electronic absorption spectra from samples of compound **1** in solid state (top) and in aqueous solution (bottom); (**b**) SEM-EDAX spectrum for compound **1**.

Compound **1** was prepared from a mixture of Gd_2_O_3_ and thymine, which reacted in an aqueous suspension acidulated with perchloric acid, and was stirred at 60 °C for 1 h. The resulting solution was left to evaporate at room temperature for 1 week, thus generating colorless parallelepipeds of **1**. Once synthesized, **1** is air-stable over a period of several days. In order to study its stability further, electronic absorption spectra of **1** were collected both in solid state and in aqueous solution (Figure 2 and Figure S2). Remarkably, the two spectra show the same absorption bands and different ones to that of the free thymine ligand (Figure S2), with this fact indicating the stability of **1** in aqueous solution (Figure 2a). SEM-EDAX analysis gave a molar ratio of 1:3 for the Gd/Cl relation in **1** (Figure 2b).

### 2.2. IR Spectroscopy

The infrared spectra of **1** and free thymine ligand are given in Figure S3. The infrared spectrum of thymine has been studied in detail in previous works [21,22], nevertheless, it has been included here just for comparison. The most interesting feature that is observed are the main vibrational bands associated to the two C=O groups, whose values for the free thymine ligand are 1735 and 1679 cm^−1^, respectively [21,22]. For **1**, these vibrational bands are observed at 1747 and 1679 cm^−1^ (Figure S3), which is consistent with the coordination of the ligand through of only one C=O group [23].

The main vibrational bands associated to perchlorate anion are observed at 1145, 1112, and 1089 cm^−1^, which indicate the presence of this anion counterbalancing the positive charges of the cationic [Gd(thy)_2_(H_2_O)_6_]^3+^ complex of **1** (Figure S3).

### 2.3. Description of the Crystal Structure

Crystal data and structure refinement parameters are summarized in Table 1. Compound **1** crystallizes in the monoclinic system with space group *P*2_1_/c. Its crystal structure is made up of [Gd(thy)_2_(H_2_O)_6_]^3+^ cations, ClO_4_^−^ anions, and water molecules of crystallization, which are held together mainly by electrostatic forces and H-bonding interactions. A [Gd(thy)_2_(H_2_O)_6_]^3+^ cation, three ClO_4_^−^ anions, and two non-coordinating water molecules are present in the asymmetric unit of **1**.

The Gd^III^ ion in **1** is eight-coordinate and bonded to eight oxygen atoms, two oxygen atoms from carbonyl groups of two neutral thymine ligands, and six oxygen atoms of six water molecules (Figure 1). Given the neutral nature of the thymine molecule, coordination through the O(1) and O(3) atoms is expected (Figures S1 and S4). The average value of the Gd-O bond lengths [2.381(1) Å] is somewhat shorter than that of the Gd-O_water_ bond lengths [2.390(1) Å] (Table S1). The O-Gd-O bond angles show values covering the range of 70.24(6)–148.57(7)°. These values are in agreement with those reported for other species with a similar Gd^III^ environment [24,25,26]. In **1**, the thymine molecules are planar and form an angle between them of ca. 7.8(1)° with an intramolecular thymine-thymine separation of ca. 4.08 Å, which corresponds to the O(1)···O(3) distance. The C−C, C−N, and C−O bond lengths agree with those found in the literature for the thymine molecule (Table S1) [27].

**Figure 3:**
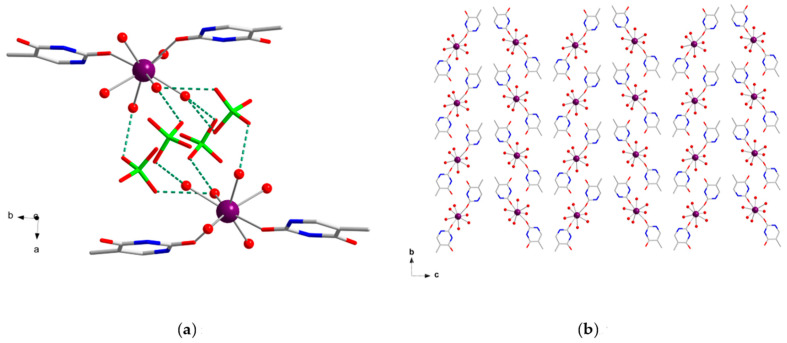
(**a**) View along the crystallographic *c* axis of the H-bonding interactions (green dashed lines) between perchlorate anions (capped sticks model) and [Gd(thy)_2_(H_2_O)_6_]^3+^ cations in **1**. For the sake of clarity, H atoms are omitted and only coordinated water molecules are shown. Color code: purple, Gd; green, Cl; red, O; blue, N; grey, C; (**b**) View along the crystallographic *a* axis of the herringbone type structure of **1**. H atoms, perchlorate anions, and non-coordinating water molecules have been omitted for clarity. Color code: purple, Gd; red, O; blue, N; grey, C.

In the crystal packing of **1**, adjacent [Gd(thy)_2_(H_2_O)_6_]^3+^ cations are connected through H-bonding interactions that occur between thymine pairs, linking them into chains [N(3)···O(4a) = 2.763(3) Å and O(2)···N(10a) = 2.785(3) Å; (a) = x, y − 1, z], which grow along the *b* crystallographic axis (Figure S5). These chains are separated from each other through perchlorate anions, which interact with coordinated water molecules by means of H-bonding interactions involving the following set of atoms: O(3w)···O(11b) [2.944(3) Å; (b) = −x + 1, −y + 1, −z + 1], O(4w)···O(10b) [2.870(3) Å], O(4w)···O(15c) [2.847(3) Å; (c) = −x + 1, y − 1/2, −z + 3/2], and O(5w)···O(12) [2.713(3) Å] (Figure 3). The chains based on [Gd(thy)_2_(H_2_O)_6_]^3+^ cations are arranged forming a herringbone type structure, generating thymine planes that exhibit an angle of ca. 64(1)° (Figure 3b). The shortest Gd···Gd separation is 8.256(1) Å [Gd(1)···Gd(1d) distance; (d) = -x, −y + 1, −z + 1]. A supramolecular network is generated by additional H-bonding interactions involving coordinated and non-coordinated water molecules, along with weaker C-H···O interactions [the average value of the C···O distance being ca. 3.34 Å for C(13)···O(16), C(6)···O(5d) and C(13)···O(11e); (e) = −x + 1, y + 1/2, −z + 3/2], which contribute to stabilizing the crystal structure in **1** (Table 2).

To further analyze the coordination environment and geometry of the Gd^III^ ion in **1**, the SHAPE program was used [28]. In **1**, the single Gd^III^ ion exhibits a coordination number (CN) equal to 8. For Gd(1), a value of 0.307 was obtained and associated with a square antiprism (SAPR) geometry, the next and closer computed value being 1.733, which was assigned to a biaugmented trigonal prism (BTPR) geometry (Table 3). Hence, these calculated values allow us to assign the D_4d_ symmetry to the Gd(1) ion in complex **1** (Table 3).

### 2.4. Analysis of the Hirshfeld Surfaces

**Figure 4:**
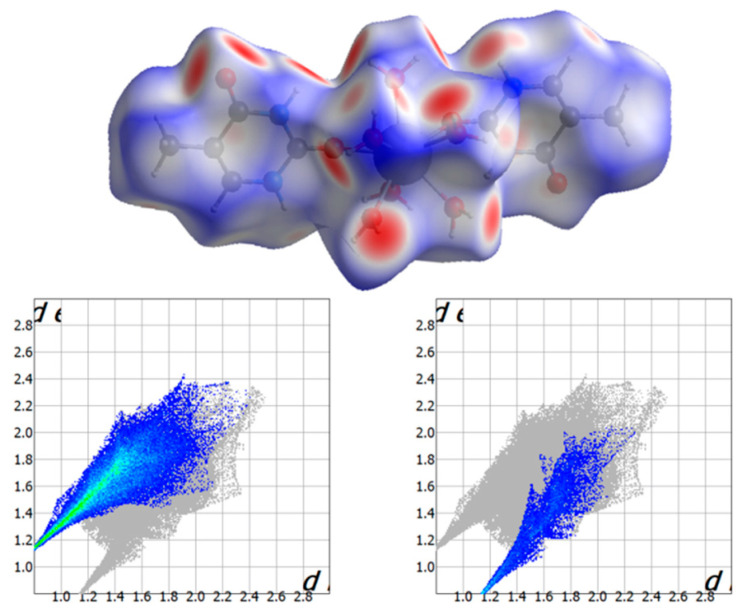
(**Top**) Hirshfeld surface mapped with *d*_norm_ function for **1**; (**bottom**) Fingerprint plots for **1**; (**bottom**, **left**) Intermolecular O···H contacts between non-coordinated and coordinated water molecules are highlighted from the full fingerprint; (**bottom**, **right**) Non-coordinated water molecules and thymine carbonyl groups are highlighted from the full fingerprint.

Hirshfeld surfaces of the cationic [Gd(thy)_2_(H_2_O)_6_]^3+^ complex were calculated, and its closer intermolecular interactions were analyzed through the *CrystalExplorer* program [29,30]. These surfaces were mapped taking into account the distance from a point on the surface to the nearest atom outside (*d*_e_) and inside (*d*_i_) the surface. To overcome limitations related to the size of atoms, a normalized contact distance (*d*_norm_) was also considered [29,30]. For compound **1**, Hirshfeld surfaces are shown in Figure 4 and Figure S6, where the shorter contacts are displayed using red color [31]. Intermolecular O···H contacts between water molecules and between water molecules and carbonyl groups of the thymine molecules are the main interactions detected on the Hirshfeld surface (Figure 4 and Figure S6). The most important O···H contacts are those involving H-bonds between non-coordinated and coordinated water molecules, which are approximately 48% of the complete fingerprint plot, whereas the O···H interactions involving non-coordinated water molecules and carbonyl groups are highlighted from the full fingerprint as ca. 19% of the plot. Finally, additional N···H contacts involving non-coordinated water molecules and N-H groups of the thymine molecules only cover approximately 2% of the fingerprint plot (Figure 4).

### 2.5. Magnetic Properties

**Figure 5:**
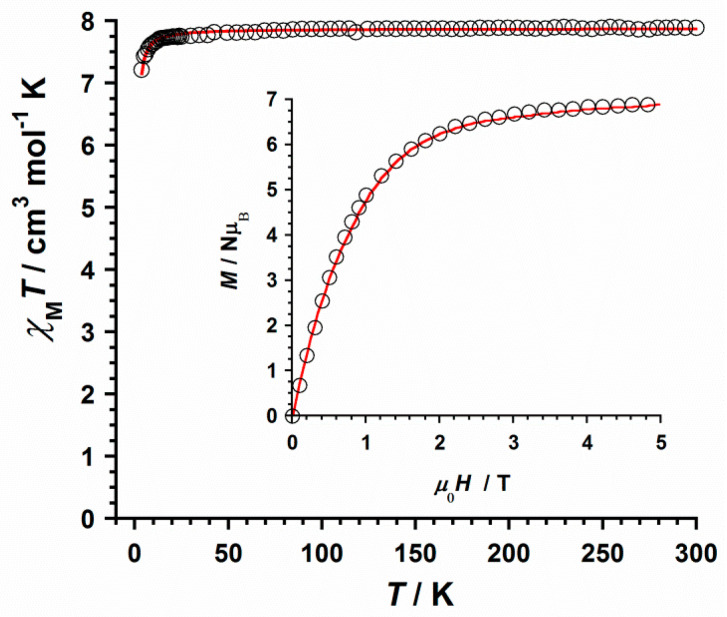
χ_M_*T* vs. *T* plot obtained for compound **1**. The inset shows the field dependence of the molar magnetization (*M*) plot at 2.0 K for **1**. The solid red lines represent the best fit of the experimental data.

Dc magnetic susceptibility measurements were carried out on a freshly prepared microcrystalline sample of **1** in the 2–300 K temperature range and under an external magnetic field of 0.5 T. The χ_M_*T* vs. *T* plot (χ_M_ being the molar magnetic susceptibility per Gd^III^ ion) for compound **1** is given in Figure 5. At room temperature, the χ_M_*T* value is ca. 7.89 cm^3^mol^−1^K, which is very close to that expected for a magnetically isolated Gd^III^ ion (4f^7^ ion with *g*_Gd_ = 2.0, S_Gd_ = 7/2 and L_Gd_ = 0), that is, 7.88 cm^3^mol^−1^K [26,32]. Upon cooling, the χ_M_*T* value approximately follows the Curie law to ca. 25 K with decreasing temperature, before χ_M_*T* decreases, reaching a minimum value of ca. 7.21 cm^3^mol^−1^ K at 2 K. The decrease of the χ_M_*T* value observed for complex **1** would likely be assignable to intermolecular interactions and/or very small zero-field splitting (ZFS) effects [32].

To analyze the magnetic behavior and fit the experimental data of the χ_M_*T* vs. *T* plot for complex **1**, the theoretical expression for the magnetic susceptibility of a single and isotropic S_Gd_ = 7/2 center was used [χ_M_ = (Nμ_B_^2^*g*_Gd_^2^/3k_B_)S_Gd_(S_Gd_ + 1)/(*T* − Θ)] [25]. Due to possible intermolecular interactions that can take place in **1**, a Θ parameter was also included in this expression. The best least-squares fit gave the parameters *g*_Gd_ = 2.008 (1) and Θ = −0.039 (1) K with *R* = 1.9 × 10^−5^ for **1** {*R* being the agreement factor defined as Σ_i_[(χ_M_*T*)_i_^obs^ − (χ_M_*T*)_i_^calcd^]^2^/[(χ_M_*T*)_i_^obs^]^2^}.

Field dependence of the molar magnetization (*M*) plot for **1** at 2 K is given in the inset of Figure 5. This plot exhibits a continuous increase of *M* with the applied magnetic field, showing a maximum value of *M* obtained for **1** (ca. 6.92 μ_B_) at 5.0 T, which is as expected for a mononuclear Gd^III^ complex [25]. The experimental data of the *M* vs. *H* plot were close to the Brillouin curve generated, with values of *g* and S of 2.0 and 7/2, respectively (Figure 5) [32].

### 2.6. MR Imaging Phantom Studies

**Figure 6:**
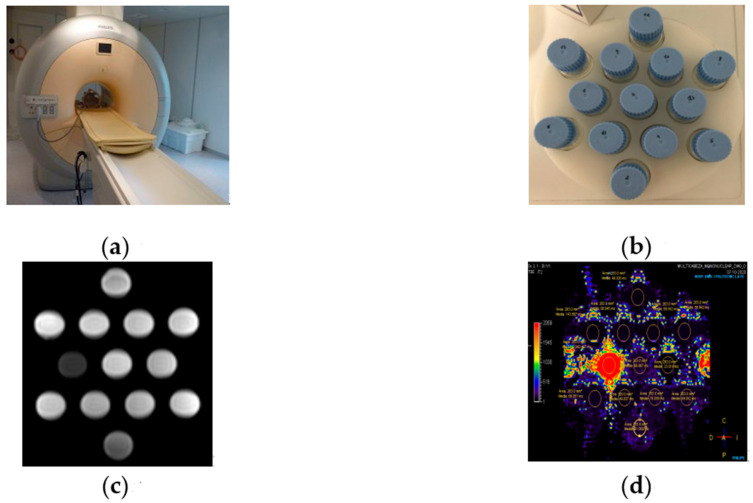
(**a**) MR imaging scanner (Philips Achieva 3T); (**b**) Samples of **1** prepared in physiological serum with concentrations covering the range of 0.0–3.2 mM; (**c**) MR images of the tube phantoms of **1**; (**d**) T_2_ parametric map analysis.

The relaxometric properties of compound **1** as a potential high-field MR imaging contrast agent were evaluated [33]. Thirteen samples of **1** were prepared in physiological serum with concentrations covering the range of 0.0–3.2 mM and were measured on a clinical MR scanner (Achieva 3T TX, Philips Healthcare, Best, The Netherlands) by using the volumetric head eight channels SENSE coil (Figure 6 and Figure S7).

**Figure 7:**
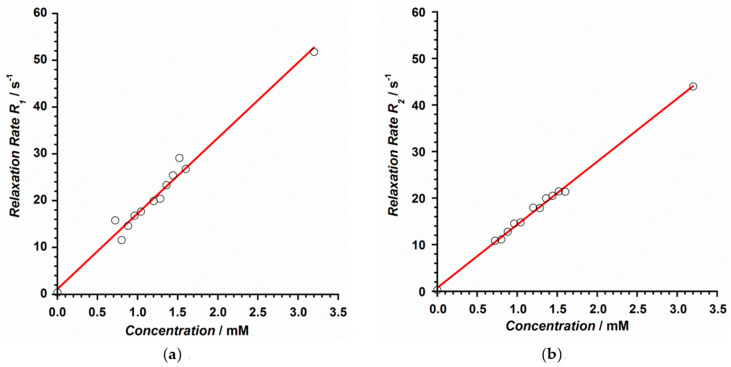
Relaxation rate vs. contrast concentration plot obtained for the relaxivities *r*_1_ (**a**) and *r*_2_ (**b**) of compound **1**. The red line represents the best linear fit of the experimental data.

**Figure 8:**
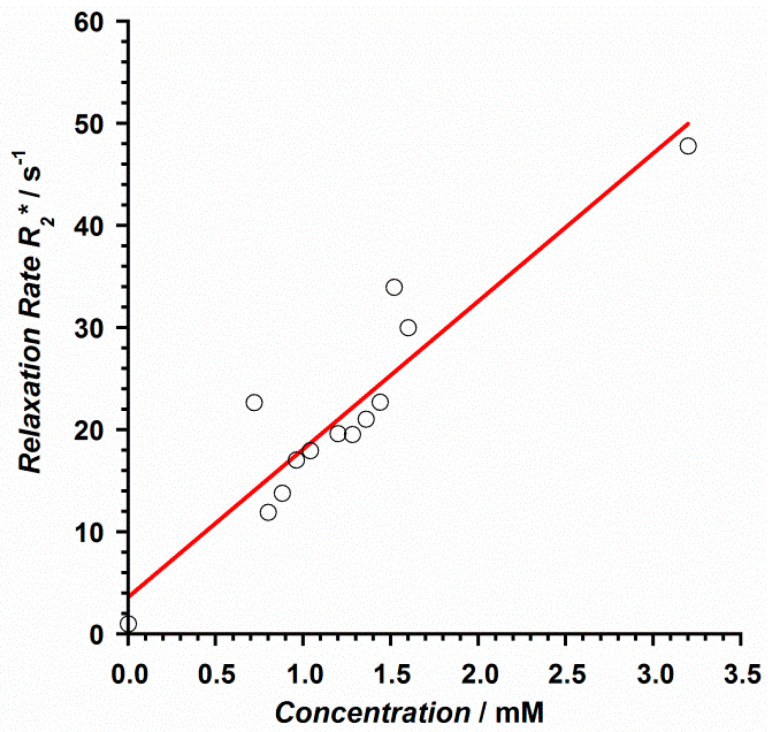
Relaxation rate vs. contrast concentration plot obtained for the relaxivity *r*_2_* of compound **1**. The red line represents the best linear fit of the experimental data.

The relaxation rate, denoted by *R* (and expressed in s^−1^), was obtained for each concentration through the calculation of the corresponding relaxation time T. *R*_1_ was obtained by calculating the T_1_ time from FFE sequences with 2°, 5°, 10°, 15°, 25°, and 45° flip angles, whereas *r*_2_ and *r*_2_* values were obtained after calculating T_2_ and T_2_* relaxation times from TSE and GRE sequences with 32 echo times each, TE_1_ = 10 ms, ∆TE = 10 ms and TE1 = 0.9 ms, ∆TE = 0.7 ms, respectively [34]. Thus, the longitudinal relaxivity (*r*_1_) of **1** at 3 T was determined to be 16.1 mM^−1^s^−1^, whereas the transversal relaxivities *r*_2_ and *r*_2_* values were 13.5 and 14.5 mM^−1^s^−1^, respectively (Figure 7 and Figure 8). These results show relaxivity values for **1** that are much higher than those of commercial MR imaging contrast agents currently employed on 3 T equipment [33], such as Magnevist, Gadovist, Prohance, Multihance, Dotarem, and Omniscan, among others [34], which makes **1** potentially useful. The fact that the Gd^III^ ion in **1** exhibits a higher number of coordinate water molecules than the typical commercial contrast agents would enhance, at least in part, the common relaxivity values reported for these commercial contrast agents [34].

## 3. Materials and Methods

### 3.1. Reagents and Instruments

All of the manipulations were performed under aerobic conditions. All the commercial chemicals were used as received. CAUTION: Although no problems were encountered in this work, care should be taken when using the potentially explosive perchlorate anion (ClO_4_^−^).

Elemental analyses (C, H, N) and X-ray microanalysis were performed by the Central Service for the Support to Experimental Research (SCSIE) at the University of Valencia. Scanning electron microscopy (SEM) images and results were obtained from a Hitachi S-4800 field emission scanning electron microscope (Hitachi Ltd., Tokyo, Japan). The electronic absorption spectra of **1** and thymine were measured at room temperature in a Jasco V-670 UV-vis-NIR spectrophotometer (Jasco Ltd., Tokyo, Japan) in the range of 600 to 1400 nm. Infrared spectra (IR) of **1** and thymine were recorded with a PerkinElmer Spectrum 65 FT-IR spectrometer (PerkinElmer Inc., Waltham, USA) in the range of 400 to 4000 cm^−1^. Variable-temperature, solid-state direct current (DC) magnetic susceptibility data down to 2.0 K were collected on a Quantum Design MPMS-XL SQUID magnetometer (Quantum Design, Inc., San Diego, CA, USA) equipped with a 5 T DC magnet. The experimental magnetic data were corrected for the diamagnetic contributions of the constituent atoms and also for the sample holder. MR data were collected on a Philips Achieva 3T clinical scanner (Philips Healthcare, Best, The Netherlands) with a volumetric head eight channels SENSE coil at La Fe University and Polytechnic Hospital.

### 3.2. Preparation of Compound

Synthesis of [Gd(thy)_2_(H_2_O)_6_](ClO_4_)_3_·2H_2_O (1)

A mixture of Gd_2_O_3_ (0.091 g, 0.25 mmol) and thymine (0.063 g, 0.50 mmol) in an aqueous suspension (5 mL) was acidulated with perchloric acid (1.0 mL, 2 M) and was stirred and heated at 60 °C for 1 h. The resulting solution was left to evaporate at room temperature for 1 week. Colorless parallelepipeds were obtained and were suitable for single-crystal X-ray diffraction studies. Yield: ca. 45%. Anal. Calcd. for C_10_H_24_N_4_O_22_Cl_3_Gd: C, 14.7; H, 3.0; N, 6.9. Found: C, 14.5; H, 3.0; N, 6.8. SEM-EDAX: a molar ratio of 1:3 for Gd/Cl was found for **1**. IR (KBr pellet/cm^−1^): peaks were observed at 3399 (br), 3218 (m), 3063 (m), 2928 (m), 2813 (m), 1747 (s), 1679 (vs), 1635 (m), 1483 (w), 1449 (m), 1427 (w), 1383 (m), 1246 (m), 1205 (m), 1145 (vs), 1112 (vs), 1089 (vs), 935 (m), 835 (m), 815 (m), 760 (m), 744 (m), 627 (vs), 560 (m), 477 (m), 432 (m).

### 3.3. X-ray Data Collection and Structure Refinement

X-ray diffraction data from a single crystal of **1** with dimensions 0.34 × 0.12 × 0.05 mm^3^ was collected on a Bruker D8 Venture diffractometer (Bruker, Billerica, MA, USA) with graphite-monochromated Mo-K*_α_* radiation (λ = 0.71073 Å). The structures were solved by standard direct methods and subsequently completed by Fourier recycling by using the SHELXTL software packages. The obtained model was refined with version 2018/1 of SHELXL against *F*^2^ on all data by full-matrix least squares [35]. All non-hydrogen atoms were anisotropically refined, whereas the hydrogen atoms of the thymine molecules were set in calculated positions and refined isotropically by using the riding model. The graphical manipulations were performed with the DIAMOND program [36]. The CCDC code for **1** is 2009091.

## 4. Conclusions

In summary, the preparation, crystal structure, magnetic properties, and MR imaging phantom studies of a novel Gd^III^ complex based on the thymine nucleobase, of formula [Gd(thy)_2_(H_2_O)_6_](ClO_4_)_3_·2H_2_O (**1**) [thy = thymine], have been reported. **1** displays the first reported crystal structure based on gadolinium and thymine. Furthermore, **1** exhibits high relaxivity values and, therefore, can be considered a suitable candidate for further developments and MR analysis. Given that most of the thymine-containing complexes have been reported with this ligand in the form of thyminate anion, we presented in this work a singular strategy to prepare lanthanide compounds where the thymine molecule acts as a neutral ligand toward the metal ion. Further investigations dealing with the synthesis and characterization of this type of lanthanide complexes are now in progress in our group, incorporating other 4f ions and similar biomolecules.

## Figures and Tables

**Table 1:** Summary of the crystal data and structure refinement parameters for **1**.

Compound	1
CCDC	2009091
Formula	C_10_H_28_N_4_O_24_Cl_3_Gd
*M*_r_/g mol^−1^	851.96
Crystal system	Monoclinic
Space group	*P*2_1_*/c*
*a*/Å	13.192 (1)
*b*/Å	9.901 (1)
*c*/Å	20.985 (1)
*α*/°	90
*β*/°	95.18 (1)
*γ*/°	90
*V*/Å^3^	2729.8 (2)
*Z*	4
*D*_c_/g cm^−3^	2.073
μ (Mo − K*_α_*)/mm^−1^	2.832
Goodness-of-fit on *F*^2^	1.081
*R*_1_ [*I* > 2*σ* (*I*)]/all	0.0280/0.0325
*wR*_2_ [*I* > 2*σ* (*I*)]/all	0.0703/0.0733

**Table 2:** Selected hydrogen-bonding interactions in **1** ^a^.

D-H·A	D-H/Å	H·A/Å	D·A/Å	(DHA)/°
N(1)-H(1)···O(8f)	0.880	2.12(1)	2.982(1)	165.4(1)
N(3)-H(3)···O(4a)	0.880	1.89(1)	2.763(1)	175.6(1)
N(10)-H(10)···O(2g)	0.880	1.92(1)	2.785(1)	168.7(1)
O(1w)-H(1wA)···O(7)	0.951	2.05(1)	2.986(1)	168.4(1)
O(1w)-H(1wB)···O(4a)	0.953	1.77(1)	2.677(1)	159.1(1)
O(2w)-H(2wA)···O(2g)	0.948	1.75(1)	2.691(1)	169.2(1)
O(3w)-H(3wA)···O(11b)	0.953	2.07(1)	2.944(1)	151.0(1)
O(3w)-H(3wB)···O(7wf)	0.950	1.76(1)	2.660(1)	156.0(1)
O(4w)-H(4wA)···O(10b)	0.951	2.01(1)	2.870(1)	149.4(1)
O(4w)-H(4wB)···O(15c)	0.953	1.944(1)	2.847(1)	157.4(1)
O(5w)-H(5wA)···O(12)	0.950	1.77(1)	2.713(1)	169.2(1)
O(5w)-H(5wB)···O(14)	0.950	1.87(1)	2.810(1)	169.1(1)
O(6w)-H(6wA)···O(9h)	0.951	2.27(1)	2.939(1)	126.9(1)
O(6w)-H(6wA)···O(7wf)	0.951	2.31(1)	2.986(1)	127.4(1)
O(6w)-H(6wB)···O(8w)	0.953	1.75(1)	2.694(1)	171.9(1)
O(7w)-H(7wB)···O(8wc)	0.961	1.91(1)	2.896(1)	172.5(1)
O(8w)-H(8wA)···O(13)	0.954	1.91(1)	2.855(1)	170.8(1)
O(8w)-H(8wB)···O(15e)	0.953	1.92(1)	2.846(1)	163.9(1)

^a^ Symmetry codes: (a) = x, y − 1, z; (b) = −x + 1, −y + 1, −z + 1; (c) = −x + 1, y − 1/2, −z + 3/2; (e) = −x + 1, y + 1/2, −z + 3/2; (f) = x, −y + 3/2, z − 1/2; (g) = x, y + 1, z; (h) = −x + 1, −y + 2, −z + 1.

**Table 3:** Selected values for possible geometries with coordination number (CN) equal to 8 obtained through the SHAPE program and from the structural parameters of complex **1** ^a^.

HPY	HBPY	CU	SAPR	TDD	JGBF	JETBPY	BTPR	JSD	TT
23.392	16.322	9.171	0.307	1.913	15.656	28.471	1.733	4.830	10.015

^a^ HPY: heptagonal pyramid (C_7v_); HBPY: hexagonal bipyramid (D_6h_); CU: cube (Oh); SAPR: square antiprism (D_4d_); TDD: triangular dodecahedron (D_2d_); JGBF: Johnson gyrobifastigium (D_2d_); JETBPY: Johnson elongated triangular bipyramid (D_3h_); BTPR: biaugmented trigonal prism (C_2v_); JSD: snub disphenoid (D_2d_); TT: triakis tetrahedron (Td).

## Data Availability

The reported data are available on request from the corresponding author.

## Supplementary Materials

The following are available online at https://www.mdpi.com/article/10.3390/ijms22094586/s1, X-ray crystallographic data in CIF format for compound **1**, Figures S1–S7, and Table S1.
